# Intestinal microbiota profile and inflammation in patients undergoing hemodialysis: a comparison between the Southern and Southeastern regions of Brazil

**DOI:** 10.1590/2175-8239-JBN-2025-0097en

**Published:** 2026-06-01

**Authors:** Bruna R. Paiva, Júnia Schultz, Fluvio Modolon, Jessyca S. de Brito, Marcelo Ribeiro-Alves, Alexandre Soares Rosado, Ludmila F.M.F. Cardozo, Denise Mafra

**Affiliations:** 1 Universidade Federal Fluminense, Programa de Pós-Graduação em Ciências Cardiovasculares, Niterói, RJ, Brazil.; 2 King Abdullah University of Science and Technology, Division of Biological and Environmental Science and Engineering, Bioscience Program, Thuwal, Saudi Arabia.; 3 Universidade de São Paulo, Instituto Oceanográfico, São Paulo, SP, Brazil.; 4 Universidade Federal do Rio de Janeiro, Programa de Pós-Graduação em Ciências Biológicas – Fisiologia, Rio de Janeiro, RJ, Brazil.; 5 Fundação Oswaldo Cruz, Instituto Nacional de Infectologia Evandro Chagas, Centro de Pesquisa Clínica em HIV/Aids, Rio de Janeiro, RJ, Brazil.; 6 Universidade Federal Fluminense, Programa de Pós-Graduação em Ciências da Nutrição, Niterói, RJ, Brazil.; 7 Universidade Federal Fluminense, Programa de Pós-Graduação em Ciências Médicas, Niterói, RJ, Brazil.

**Keywords:** Renal Insufficiency, Chronic, Microbioma Gastrointestinal, Renal Dialysis, Geography

## Abstract

**Introduction:**

Exogenous lifestyle factors, such as different cultures, diets, and geo­graphic location, can alter the microbiota in patients with chronic kidney disease (CKD), which is closely related to inflammation. However, few studies have examined how these factors influence the composition of the microbiota. Thus, the objective of this study was to characterize and compare the intestinal microbiota profile and inflammation in CKD patients undergoing hemodialysis (HD) in the Southern and Southeastern regions of Brazil.

**Methods:**

Blood and stool samples were obtained from two groups of HD patients: one from the city of Blumenau (Southern region) and the other from the city of Rio de Janeiro (Southeastern region). Fecal DNA was extracted, and the V4 region of the bacterial 16S ribosomal RNA gene was sequenced. The fecal microbiome was analyzed using bioinformatic tools. Plasma concentrations of IL-6 and TNF-α were evaluated by ELISA.

**Results:**

Thirty patients were included in the study, with 14 individuals residing in the Southern region (group S) [50% male, 58 (13.5) years of age] and 16 individuals residing in the Southeastern region (group SE) [47.1% male, 57 (19) years of age]. The α- and β-diversity indices of the intestinal microbiota did not differ significantly between the groups. However, patients from the Southern region had higher plasma TNF-α (p = 0.008) and IL-6 (p = 0.003) levels than those from the Southeastern region.

**Conclusion:**

Although HD patients with CKD residing in the Southern and Southeastern regions present similar intestinal microbial patterns, patients from the Southern region had higher concentrations of inflammatory markers.

## Introduction

Scientific studies on gut microbiota have been gaining attention, and this is no different in nephrology, since the imbalance of the gut microbiota in patients with chronic kidney disease (CKD) has been identified as a new factor related to inflammation and oxidative stress. These conditions contribute to the development and progression of CKD, as well as to cardiovascular diseases (CVD)^1,2^.

A balanced composition of bacteria, fungi, archaea, and viruses maintains a homeostatic relationship with its host. When the proportion of opportunistic bacteria increases and bacterial diversity declines, an imbalance of the intestinal microbiota, known as intestinal dysbiosis, occurs^3^. Several factors contribute to the intestinal microbiota imbalance in patients with CKD, including uremia. Elevated plasma urea levels cause an influx of this molecule into the intestinal lumen. Bacteria that express the enzyme urease hydrolyze urea into ammonia, increasing the intestinal pH and promoting changes in the biochemical environment, favoring the growth of certain bacteria at the expense of others^4^. Furthermore, this toxicity causes the rupture of tight junctions, allowing the translocation of pathogen-associated molecular patterns (PAMPs), such as lipopolysaccharides (LPS), components of the membrane of Gram-negative bacteria^5^. In addition, factors such as sedentary lifestyle, stress, alcohol consumption, diet, food additives and pesticides, antibiotic use, genetics, edema, constipation, changes in nutritional status, and certain diseases alter the gut microbiota of these patients^6,7^. The sociocultural context in which the individual is embedded also contributes to the characterization of the gut microbiota; thus, interindividual variation in this microbiome typically follows characteristic patterns among individuals residing in the same geographic location^8,9^.

All these factors can alter the microbiota of patients with CKD, and this alteration can have several consequences, including an increase in the inflammatory process. This occurs because both components of the microbiota and the toxins they produce can reach the bloodstream and activate inflammatory factors. This leads to increased production of pro-inflammatory cytokines, such as tumor necrosis factor-α (TNF-α) and interleukin-6 (IL-6), by activated monocytes and endothelial cells^10^.

Research indicates that diet and lifestyle are the most influential factors in characterizing the gut microbiota and are closely related to the sociocultural context and geographic location of these indi­viduals^11,12^. Studies on the microbiota in patients with CKD have focused on both evaluating the overall microbial profile and on interventions aimed at mitigating dysbiosis, using prebiotics, probiotics, or synbiotics^13^. However, it is also necessary to study the microbiota profile across markedly different geographic and cultural settings, such as the Southern and Southeastern regions of Brazil.

Given that geographic location and sociocultural context significantly influence the composition of the microbiota, particularly through environmental differences, regional dietary patterns, lifestyle habits, and variations in dialysis-related care, it is important to investigate how these factors affect patients with CKD. Factors such as the availability and type of food consumed, regional culinary practices, and potential differences in care across dialysis units can modify both the microbial profile and the inflammatory state of these individuals. Due to the scarcity of studies evaluating these specific aspects in the Brazilian context, especially in culturally and environmentally distinct regions such as the South and the Southeast, this investigation was deemed necessary. Its primary objective was to characterize and compare inflammation and the intestinal microbial profile of CKD patients undergoing HD in these two regions.

## Methods

### Study Design

This cross-sectional study included patients with CKD undergoing hemodialysis (HD) (three sessions per week, each lasting 4 hours) residing in the Southern (Blumenau) and Southeastern (Rio de Janeiro) regions of Brazil. The study included men and women aged 18 to 75 years who had been undergoing HD for at least six months and were treated by the Brazilian Unified Health System (SUS). The study excluded pregnant women, transplant recipients, and patients with liver, autoimmune, or infectious diseases, cancer, or acquired immunodeficiency syndrome (AIDS). Patients using catabolic drugs or prebiotic, probiotic, or synbiotic supplements, as well as those who had used antibiotics or anti-inflammatory drugs in the three months prior to the start of the study, were also excluded.

The present study was approved by the Research Ethics Committee of the Faculty of Medicine of UFF-Niterói, Rio de Janeiro, Brazil (CAAE 47703315. 6.0000.5243 and 03219618.3.0000.5243), and all patients gave their informed consent before study initiation.

The demographic and clinical variables used in the study were obtained directly from medical records, thereby ensuring the standardization of information and fidelity to the original documen­tation by the care team. This procedure enabled the collection of complete and consistent data on participants’ profiles, ensuring the quality and reliability of the information used in the analyses.

### Analysis of Food Intake and Assessment of Nutritional Status

Dietary intake was assessed using a three-day dietary recall, covering two weekdays (one with dialysis and one without) and one weekend day. Energy and macronutrient intake analyses were calculated using NutWin® software. It is important to note that patients followed individualized dietary prescriptions provided at the dialysis clinics.

Nutritional status was assessed using body mass index (BMI), defined as weight in kilograms divided by height in meters squared, following the classification proposed by the World Health Organization^14^.

For the evaluation of the dialysis dose, Kt/V was calculated using the Daugirdas formula (second generation), which estimates hemodialysis efficiency from pre- and post-dialysis urea concentrations, treatment time, and body weight variation during the session^15^.

### Sample Processing and Analytical Procedures

Blood was collected at a single time point in the morning, after fasting, before the start of the dialysis procedure, and immediately after arteriovenous fistula (AVF) puncture. Blood was collected in Vacutainer® tubes containing ethylenediaminetetraacetic acid (EDTA – 1.0 mg/mL) as an anticoagulant. Plasma was separated (15 min, 3500 rpm, 4 °C) and stored at -80 °C until analysis.

#### Routine biochemical parameters and inflammatory cytokines

Serum levels of biochemical parameters such as urea, calcium, albumin, glucose, phosphorus, potassium, total cholesterol, high-density lipoprotein (HDL), triglycerides, and C-reactive protein (CRP) were analyzed using commercial BioClin® kits with an automated biochemical analyzer, according to the manufacturer’s instructions. Low-density lipoprotein (LDL) values were calculated with the Friedewald formula (1972): LDL = (TC – HDL) – (TG/5)^16^.

For measuring plasma concentrations of the inflammatory cytokine IL-6 and TNF-α, commercial enzyme-linked immunosorbent assay (ELISA) kits were used. IL-6 and TNF-α concentrations were measured within the range of 31–2000 pg/mL using specific quantitative sandwich ELISA kits (PeproTech, Inc.).

#### Lipid peroxidation

Lipid peroxidation was used as a marker of oxidative stress, assessed by measuring thiobarbituric acid reactive substances (TBARS) using the modified Ohkawa method. Samples were diluted with thiobarbituric acid (0.6% w/v), sodium dodecyl sulfate (SDS) (8.1% w/v), and phosphoric acid (1% w/v) and subsequently heated to 95 °C for 60 min. Microtubes were centrifuged at 4000 rpm for 20 min at 20 °C; the supernatant was removed, and absorbance was measured by a Synergy H1M microplate reader (Biotek) at 532 nm. Plasma TBARS levels were expressed as nmol/mL.

### Sequencing and Analysis of the Gut Microbiota

Patients were previously provided with sterile stool sample collection containers and a manual containing instructions on sample collection, storage, and transport. They were instructed to keep the stool samples in the freezer overnight and transport them under controlled temperature conditions. Upon receipt by our team, the samples were stored at -20°C for subsequent analysis. DNA extraction was performed according to the manufacturer’s instructions (Quick-DNA Fecal/Soil Microbe DNA Miniprep Kit, Zymo Research). Final DNA concentrations were determined by spectrophotometric quantification using the NanoDrop 2000 (Thermo Fisher Scientific, Waltham, MA, USA).

The V4 region of the 16S rRNA gene was amplified by PCR using primers 515F 5’-GTGY CAGCMGCCGCGGTAA-3’ and 806R 5’-GGACT ACNVGGGTWTCTAAT-3’ (attached to Illumina universal tags) and subjected to thermocycling, consisting of 3 min of initial denaturation at 94 °C, followed by 32 cycles of 45 s at 94 °C, 1 min at 50 °C, and 90 s at 72 °C, with a final extension step of 10 min at 72 °C. The resulting amplicons were barcoded and sequenced on the Illumina NovaSeq PE250 platform (0.1 M raw reads per sample), according to the manufacturer’s instructions, by Novogene (California, USA). In total, 30 samples from two groups (HD and PD) were sequenced by amplicon.

Amplicon sequencing reads of the 16S rRNA gene were preprocessed using the USEARCH pipeline (v. 11). This included sample clustering, maintaining a unique sample tag in the raw file headers, followed by paired-end read assembly/fusion, primer trimming, and quality filtering. Additionally, abundances were calculated for unique sequences, and operational taxonomic units (OTUs) clustering with 97% identity was performed, followed by a noise reduction step to filter chimeras, resulting in a table of zero-radius OTUs (zOTUs). The feature and taxonomy tables, along with the metadata, were exported as phyloseq objects for further analysis in R version 4.1.2.

### Statistical Analysis

Continuous numerical baseline demographic and clinical variables were compared using the nonparametric Mann–Whitney U tests. Chi-square tests were used to compare the relative frequencies of the different levels of nominal/categorical variables. Plasma levels of the studied variables were log-transformed (base 10) when necessary, and the studied mRNA expression levels were log-transformed (base 2). Adjusted Pearson correlations were performed using residuals from linear fixed-effects models, including confounding variables (i.e., age, sex, BMI, time on dialysis, and Kt/V). Multiple linear fixed-effects models were also used to assess differences between the S and SE groups, controlling for the same set of confounding variables. The graphs present the estimated mean marginal effects and their 95% confidence intervals.

In the analysis of the gut microbiota, to enable a fair comparison between the S and SE groups, data were rarefied for downstream analyses. Rarefied data generated α-diversity indices (observed number of zOTUs and Shannon diversity). Raw zOTU data were normalized using the DESeq2 library in R^17^ and used to calculate the weighted UniFrac distance, which was visualized via principal coordinate analysis (PCoA) using the phyloseq package implemented in R. Permutational multivariate analysis of variance (PERMANOVA) was performed on the data matrix to compare the structure or composition of the microbial communities. Permutational multivariate dispersion analysis (PERMDISP) was also performed using the ‘betadisper’ function (implemented in the vegan package, version 2.6-4) with 999 permutations.

Descriptive and comparison of means analyses, as well as redundancy and Pearson correlation analyses, were performed in R, version 4.1.2, using the ‘emmeans’ (version 1.8.2) and ‘microViz’ (version 0.11.0) packages. Relationships between zOTUs and sample groups were assessed using the ‘indicspecies’ (version 1.7.14) package. All graphs and plots were generated using the ggplot2 (version 3.4.0) or TBtools^18^ packages.

## Results

**Figure 1:**
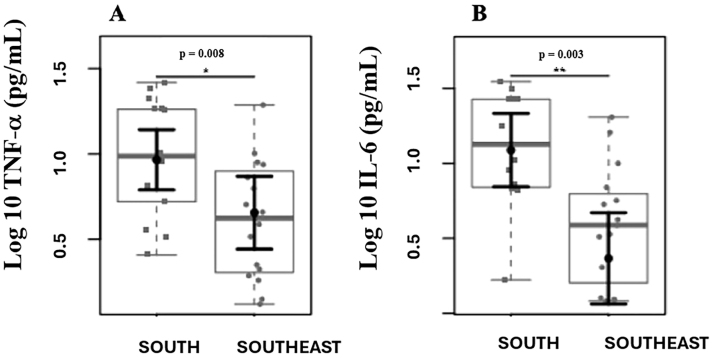
Comparison of plasma levels of inflammatory markers. Differences were observed in plasma levels of TNF-α (A) and IL-6 (B) between the Southern and Southeastern groups.

A total of 30 patients were included, with 14 in the S group and 16 in the SE group. Table 1 shows the demographic data, dietary intake, biochemical, and inflammatory parameters of the patients included in the study. Demographic and clinical parameters (age, sex, dialysis time, Kt/V, BMI) did not show statistically significant differences; energy intake (p = 0.029) and carbohydrate intake (p = 0.017) were higher in the Southeastern group. Patients from the Southern region had higher serum albumin (p = 0.011) and potassium (p = 0.011) levels. Furthermore, patients from the Southern region had higher plasma levels of inflammatory cytokines (Figure 1).

**Table 1:** Demographic, dietary, biochemical, and inflammatory parameters of patients with CKD

Parameters	Southern group (n = 14)	Southeastern group (n = 16)	p-value
**Demographic and Clinical**			
Age (years)	58 (13.5)	57 (19)	0.691
Sex [n (%)] (male/female)	7(50%)/7(50%)	8 (47.1%)/9 (52.1%)	1.000
Dialysis time (months)	40.5 (27)	68 (79)	0.142
Kt/V	1.40 (0.2)	1.42 (0.18)	0.883
BMI (kg/m^2^)	24.8 (4.57)	24.4 (4.43)	0.592
**Dietary**			
Energy (kcal/day)	1035.5 (368.7)	1499.3 (568)	**0.029**
Carbohydrate (g/day)	135.8 (46.0)	213.34 (89.8)	**0.017**
Protein (g/day)	53.7 (25.3)	74.2 (43.6)	0.161
Lipids (g/day)	35.4 (18.0)	40.3 (12.3)	0.487
Fiber (g/day)	10.2 (6.4)	13.7 (5.6)	0.207
**Biochemical**			
Glucose (mg/dL)	97 (63.5)	100 (26)	0.682
Total cholesterol (mg/dL)	163.5 (34)	153 (30)	0.706
Triglycerides (mg/dL)	159.5 (58)	114 (41)	0.108
HDL (mg/dL)	43.5 (6)	40 (8)	0.320
LDL (mg/dL)	78.4 (28)	57.0 (51)	0.511
Phosphorus (mg/dL)	5.1 (0.9)	4.6 (1.3)	0.103
Potassium (mg/dL)	4.0 (0.2)	3.7 (0.4)	**0.011**
Calcium (mg/dL)	8.0 (1.1)	8.9 (0.6)	0.062
Urea (mg/dL)	130.5 (19.7)	140 (3.5)	0.180
Albumin (mg/dL)	4.0 (0.2)	3.7 (0.4)	**0.011**
**Inflammatory**			
CRP (mg/dL)	4.7 (8.8)	6.5 (9.2)	0.580
TBARS (nmol/mL)	2.5 (2.4)	1.4 (4.1)	0.467

Abbreviations – CKD: chronic kidney disease; Kt/V: dialysis efficacy; BMI: body mass index; HDL: high-density lipoprotein; CRP: C-reactive protein; TBARS: thiobarbituric acid reactive substances. Notes – Data are presented as absolute (relative) frequencies or medians (interquartile range [IQR]); p-values were estimated using chi-square or nonparametric Mann–Whitney U tests.

Notes – The sample data distributions are presented as box-strip plots. In black, the central circle represents the average marginal effect for each group, estimated from multiple linear fixed-effects models, including the confounding variables age, sex, BMI, time on dialysis, and Kt/V. The black horizontal bars represent the 95% confidence intervals of the estimated average marginal effect per group.

### Structure and Diversity of the Gut Microbiota in CKD Patients in Different Dialysis Groups

**Figure 2:**
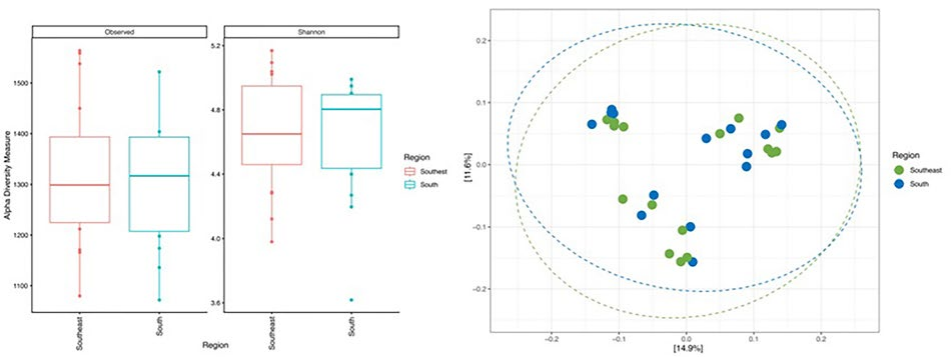
α- and β-diversity and microbial community structure of the gut microbiota of CKD patients on HD living in the Southern and Southeastern regions of Brazil.

Microbial diversity (Shannon index, Figure 2A), richness (observed number of zOTUs, Figure 2B), and microbial community structure (weighted UniFrac distance, Figure 2C) at the zOTU level of the gut microbiota of CKD patients undergoing HD residing in the Southern and Southeastern regions of Brazil did not show significant differences.

Notes – Observed zOTUs (A), Shannon index (B), and comparison of community structure (C) (weighted UniFrac distances) using principal coordinates analysis (PCoA), showing percentage variability in the microbiome caused by the groups.

**Figure 3:**
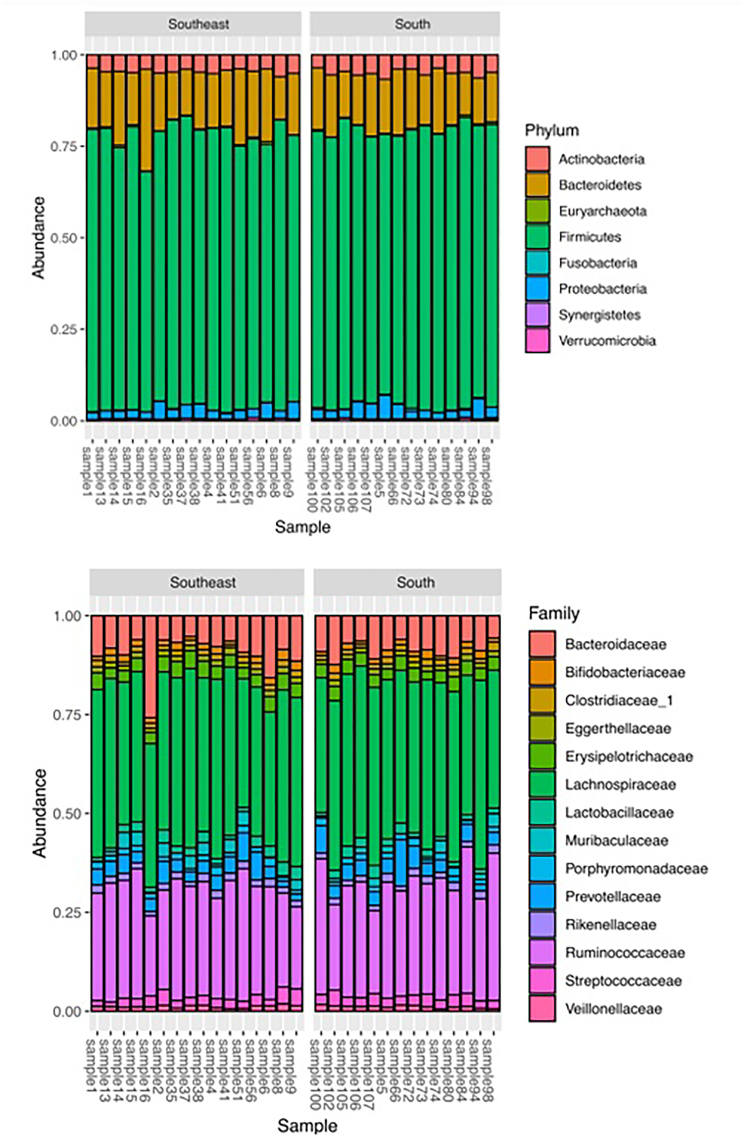
Intestinal microbial composition of CKD patients on HD living in the Southern and Southeastern regions.

Figure 3 shows the most abundant microbial taxa in the 30 samples analyzed. The phyla Firmicutes and Bacteroidetes and the families Lachnospiraceae and Ruminococcaceae were the most abundant taxa found in all samples.

Notes – Bar plots representing the most abundant phyla (A) and families (B) in each sample.

**Figure 4:**
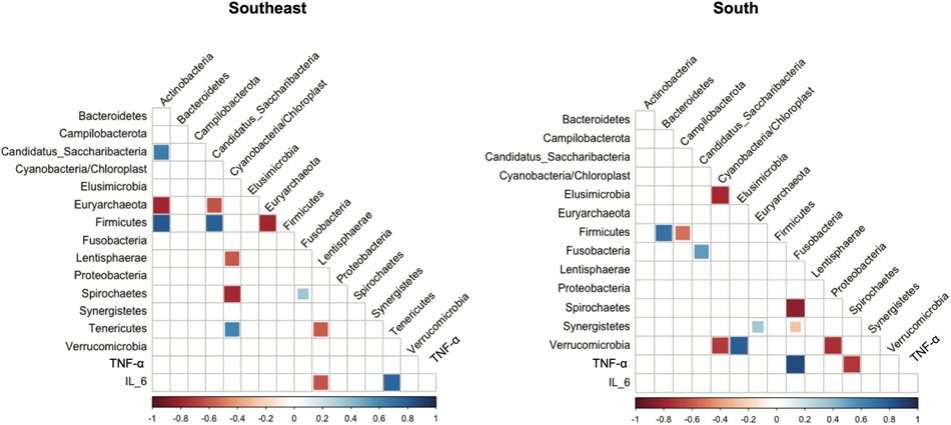
Correlogram between bacterial phyla, microbial taxa, and inflammatory markers.

The correlogram (Figure 4) represents the significant correlations (p < 0.05) between bacterial phyla and inflammatory markers (IL-6 and TNF-α). Although the α- and β-diversity analyses showed no differences in the structure of the microbial community between the two regions, distinct correlation patterns were observed. In the Southeastern group, no correlation was found between plasma TNF-α levels and any taxon analyzed; however, a negative correlation between IL-6 and Lentisphaerae and a positive correlation between IL-6 and Tenericutes were identified. In the Southern group, no correlation was observed between plasma IL-6 levels and any taxon analyzed, whereas TNF-α correlated negatively with Spirochaetae and positively with Fusobacteria.

Notes – Only significant correlations (p < 0.05) were shown in the graph: positive (blue) and negative (red) according to Pearson's linear correlation test adjusted for age, sex, and BMI, time on dialysis, and Kt/V.

## Discussion

Our study aimed to characterize and compare gut microbial patterns and inflammatory profiles among hemodialysis patients residing in the Southern and Southeastern regions of Brazil, and similar patterns were observed. However, patients from the Southern region exhibited a more pronounced inflammatory profile than those from the Southeastern region.

The Southeastern group showed higher energy intake than the Southern group, and this finding reflected increased carbohydrate consumption. However, no correlation was found with the composition of the intestinal microbiota. It is worth noting that dietary intake was assessed using a three-day dietary recall. Although this method provides information on the dietary pattern of the study population, it has limitations and therefore does not provide a reliable estimate of nutrient absorption^19^. However, it is very likely that these individuals exhibit low variability in food consumption, since our sample consisted of patients treated within the Brazilian Public Health System (SUS). This population mostly belong to lower socioeconomic strata, have less purchasing power, and have limited access to a wide range of nutriti­onally adequate foods.

Patients from the Southern group had higher serum albumin levels than those in the Southeastern group. A possible explanation for this result is differences in nutritional monitoring among these patients. Nerbass et al.^20^ reported that the Southern region had the highest frequency of free nutritional supplementation provided to dialysis patients (71%), followed by the Northeastern (55%), Northern (50%), Midwestern (49%), and Southeastern regions (40%).

In this study, we used high-throughput sequencing to compare the gut microbiota of CKD patients undergoing HD residing in the Southern and Southeastern regions of Brazil. Our aim was to elucidate whether local culture, along with dietary factors, influences gut microbial composition, while identifying potential intestinal microbial patterns and informing dietary therapy for these patients. Refuting our initial hypothesis, the gut microbiota composition in CKD patients undergoing HD residing in different regions of Brazil (Southern vs. Southeastern) did not differ. A striking bacterial pattern was observed in both groups, with a predominant abundance of the phyla Firmicutes and Bacteroidetes and the families Lachnospiraceae and Ruminococcaceae.

To date, no study has examined differences in the microbiota of CKD patients across regions of Brazil, nor has any assessed inflammatory parameters. It is important to emphasize that our study analyzed taxa at the phylum and family levels; therefore, we cannot assert the superiority of one microbial pattern over another at the genus or species taxonomic levels, since the same family may contain commensal or pathogenic bacterial lineages. Thus, studies employing metagenomic approaches and long-read amplicon sequencing should help elucidate the distribution patterns of the microbiome across different patients in the future. Furthermore, substrate availability can interfere with the metabolic pathways of intestinal bacteria, such that fecal proteolytic bacterial taxa, and therefore producers of uremic toxins, can also generate phenolic compounds, depending on the available substrate^21,22^.

Some studies report alterations in the gut microbiota in patients with CKD. The first study to report an altered gut microbiota profile in these population, with increased growth of bacteria from the genera *Streptococci, Lactobacilli*, and *Bacteroides*, was conducted in 1978. The authors hypothesized that uremia reduced intestinal transit and impaired immune function, thereby favoring the growth of certain bacteria^23^. Vaziri et al.^24^ observed that patients with CKD showed a significant difference in 190 OTUs compared with healthy individuals. These individuals exhibited a significant expansion of intestinal bacteria with enzymes involved in uremic toxin production, along with a reduction in bacterial families that produce short-chain fatty acids (SCFAs). This profile indicates an unfavorable composition, given the deleterious effects of uremic toxins and the beneficial effects of SCFAs^7,24,25^. In patients with CKD, the colon becomes the primary secretory pathway for uric acid and oxalate; this mechanism explains the expansion of uricase-producing bacterial species^26^. The microbiota of CKD patients on hemodialysis appears to differ from that of healthy controls, showing a greater abundance of the bacterial phyla Actinobacteria, Firmicutes, and Proteobacteria compared with healthy controls^24^. The gut microbial population differs between patients undergoing PD and healthy controls, with a lower abundance of *Bifidobacterium catenulatum*, *Bifidobacterium longum*, *Bifidobacterium bifidum*, *Lactobacillus plantarum*, *Lactobacillus paracasei*, and *Klebsiella pneumoniae* ^27^. Thus, CKD patients appear to have a greater abundance of bacteria that express urease, uricase, and enzymes that form indole and p-cresol^28^.

Our study found distinct correlation patterns. In the Southeastern group, a negative correlation was observed between IL-6 and Lentisphaerae, and a positive correlation between IL-6 and Tenericutes. Another study conducted by our group found a positive correlation between Fusobacteria and the uremic toxin indoxyl sulfate (IS) and a negative correlation with Lentisphaerae in HD patients^29^. Zhang et al.^30^ revealed that the phylum Lentisphaerae showed a negative correlation with the risk of sepsis. In contrast, the phylum Tenericutes was positively associated with the risk of sepsis in individuals under 75 years of age.

Furthermore, Nagu et al.^31^ detected that intestinal taxa, such as Actinobacteria, Bacteroidetes, *Escherichia coli*, Firmicutes, Proteobacteria, Tenericutes, and Verrucomicrobia, are known to play a crucial role in the pathogenesis of Alzheimer’s disease. These taxa contribute to neuroinflammation and other inflammatory processes, thereby impairing neurodevelopmental processes.

In patients from the Southern region, TNF-α values correlated positively with Fusobacteria and negatively with Spirochaetae. Some studies have demonstrated the influence of Fusobacteria on the etiology of colorectal cancer. The mechanism underlying this association is complex; however, research shows that *Fusobacterium nucleatum* promotes intestinal inflammation by stimulating the secretion of pro-inflammatory cytokines interleukin-8 (IL-8) and tumor necrosis factor (TNF) through outer membrane vesicles that activate Toll-like receptor 4 (TLR-4) and NF-κB^32,33^. In general, Fusobacteria are Gram-negative anaerobes that play a key role in oral biofilms and are involved in periodontal and extraoral diseases, with colorectal cancer being the most prominent, representing an inflammation-promoting taxon^34,35,36^.

Further studies are needed to elucidate the negative correlation observed with Spirochaetae and TNF-α, as this taxon comprises three genera that cause human infection: *Treponema* spp., which cause syphilis; *Borrelia* spp., responsible for Lyme disease; and *Leptospira*, the causative agent of leptospirosis^37^. The pathophysiology of these infections is complex, and understanding these mechanisms may help clarify our results.

A study revealed that Lyme arthritis induces an excessive pro-inflammatory immune response due to the production of IFN-γ and inadequate levels of the anti-inflammatory cytokine IL-10, promoting tissue and vascular damage, autoimmune and cytotoxic processes, as well as fibroblast proliferation and fibrosis^38^. Thus, although Spirochaetae includes species that cause inflammatory diseases, its pathophysiology may not lead to increased TNF-α levels but rather to elevations in other inflammatory cytokines, such as IFN-γ.

Overall, although our study did not identify significant differences in microbiota structure between the two regions analyzed, as indicated by α- and β-diversity metrics, distinct patterns of correlation between bacterial phyla and inflammatory markers were observed, as discussed previously. However, these associations should be interpreted with caution. Since there were no clear differences in microbial composition between the groups, the observed patterns may reflect intragroup variability or residual confounding not captured by our analyses. Thus, while the findings raise interesting hypotheses about possible interactions between the microbiota and inflammatory pathways, they do not allow the inference of causal relationships or robust biological differences between the regions. Studies with larger sample sizes, functional analyses, and broader control of potential confounders are needed to confirm or refute these observations.

There is a scarcity of comparative studies that simultaneously evaluate the gut microbiota and inflammatory markers in CKD patients on HD residing in different regions of Brazil. Although national studies have focused on characterizing dysbiosis associated with CKD and its correlations with uremic toxins, inflammation, and diet, these have been conducted predominantly in localized cohorts, without multicenter analyses that would allow the identifi­cation of geographic variation, which underscores the relevance and originality of the present study.

This study has some limitations, such as the absence of a questionnaire to assess socioeconomic variables, although we knew that 100% of our sample consisted of patients treated within the Brazilian public health system (SUS). Another important factor is the assessment of medication use; however, we were careful during patient selection, excluding individuals using catabolic drugs and prebiotic, probiotic, or synbiotic supplements, as well as those who had used antibiotics or anti-inflammatory drugs in the three months prior to blood collection.

Although our analyses did not reveal significant differences in the diversity or structure of the microbiota between the regions, the distinct regional patterns of association between bacterial taxa and inflammatory markers suggest that the environmental and dietary context may differentially influence the microbiota-inflammation interaction in hemodialysis patients. This type of information has the potential to guide the development of precision nutrition approaches, which consider not only microbial composition but also its functional interrelationships with the inflammatory state. Thus, understanding how regional profiles shape these interactions can inform more individualized strategies for modulating inflammation, offering promising avenues for interventions to improve patient prognosis.

## Conclusion

Our study found that, although hemodialysis patients with CKD residing in the Southern and Southeastern regions of Brazil exhibited similar patterns of diversity and structure in intestinal microbial communities, the Southern group showed higher concentrations of inflammatory markers (TNF-α and IL-6). It is important to emphasize that our study was pioneering in this field; therefore, further studies are needed to support our findings.

## Data Availability

The authors confirm that the data supporting the results of this study are available within the manuscript.
