# Supplementary material for: Intestinal microbiota profile and inflammation in patients undergoing hemodialysis: a comparison between the Southern and Southeastern regions of Brazil
Source: J Bras Nefrol. 2026 Jun 1;48(3):e20250097. doi: 10.1590/2175-8239-JBN-2025-0097en (PMC13225290; doi:10.1590/2175-8239-JBN-2025-0097en)
Supplement: Translation

# Translation: Perfil da microbiota intestinal e inflamação em pacientes em hemodiálise: comparação entre as regiões Sul e Sudeste do Brasil

**DOI:** 10.1590/2175-8239-JBN-2025-0097pt

**Authors:** Bruna R. Paiva, Júnia Schultz, Fluvio Modolon, Jessyca S. de Brito, Marcelo Ribeiro-Alves, Alexandre Soares Rosado, Ludmila F.M.F. Cardozo, Denise Mafra

**Affiliations:** 1 Universidade Federal Fluminense, Programa de Pós-Graduação em Ciências Cardiovasculares, Niterói, RJ, Brasil.; 2 King Abdullah University of Science and Technology, Division of Biological and Environmental Science and Engineering, Bioscience Program, Thuwal, Saudi Arabia.; 3 Universidade de São Paulo, Instituto Oceanográfico, São Paulo, SP, Brasil.; 4 Universidade Federal do Rio de Janeiro, Programa de Pós-Graduação em Ciências Biológicas – Fisiologia, Rio de Janeiro, RJ, Brasil.; 5 Fundação Oswaldo Cruz, Instituto Nacional de Infectologia Evandro Chagas, Centro de Pesquisa Clínica em HIV/Aids, Rio de Janeiro, RJ, Brasil.; 6 Universidade Federal Fluminense, Programa de Pós-Graduação em Ciências da Nutrição, Niterói, RJ, Brasil.; 7 Universidade Federal Fluminense, Programa de Pós-Graduação em Ciências Médicas, Niterói, RJ, Brasil.

**Keywords:** Insuficiência Renal Crônica, Microbioma Gastrointestinal, Diálise Renal, Geografia

## Abstract

**Introdução:**

Fatores exógenos relacionados ao estilo de vida, como culturas diferentes, alimentação e localização geográfica, podem alterar a microbiota em pacientes com doença renal crônica (DRC), a qual tem íntima relação com inflamação. No entanto, poucos estudos abordaram a influência desses fatores na alteração da composição da microbiota. Assim, o objetivo deste estudo foi caracterizar e comparar o perfil da microbiota intestinal e inflamação em pacientes com DRC em hemodiálise (HD) das regiões Sul e Sudeste do Brasil.

**Métodos:**

Foram obtidas amostras de sangue e fezes de dois grupos de pacientes em HD: um grupo da cidade de Blumenau (região Sul) e outro da cidade do Rio de Janeiro (região Sudeste). A extração de DNA fecal foi realizada, e as regiões V4 do gene de RNA ribossomal bacteriano 16S foram sequenciadas. O microbioma fecal foi analisado usando bioinformática. As concentrações plasmáticas de IL-6 e TNF-α foram avaliadas por ELISA (Peprotech®).

**Resultados:**

Trinta pacientes foram incluídos no estudo, sendo 14 indivíduos residentes da região Sul (grupo S) [50% homens, 58 (13,5) anos de idade] e 16 indivíduos residentes da região Sudeste (grupo SE) [47,1% homens, 57 (19) anos de idade]. Os índices de diversidade microbiana intestinal α e β não mostraram diferença significativa entre os grupos. No entanto, observou-se que pacientes da região Sul apresentavam maiores valores de TNF-α (p-valor = 0,008) e IL-6 (p-valor = 0,003) plasmáticos quando comparados com pacientes da região Sudeste.

**Conclusão:**

Embora pacientes com DRC em HD provenientes do Sul e Sudeste apresentem o mesmo padrão microbiano intestinal, aqueles da região Sul tiveram maiores concentrações de marcadores inflamatórios.

## Introdução

Estudos científicos sobre microbiota intestinal vêm ganhando notoriedade, e não é diferente na nefrologia, uma vez que o desequilíbrio da microbiota intestinal em pacientes com doença renal crônica (DRC) tem sido apontado como um novo fator relacionado à inflamação e ao estresse oxidativo, condições que contribuem para o desenvolvimento e a progressão da DRC, bem como para o desenvolvimento de doenças cardiovasculares (DCV)^1,2^.

A microbiota saudável, com sua composição equilibrada entre bactérias, fungos, arqueas e vírus, mantém com seu hospedeiro uma relação homeostática. Quando ocorre aumento do táxon de bactérias oportunistas e declínio da diversidade bacteriana, instala-se o quadro de desequilíbrio da microbiota intestinal, denominado disbiose intestinal^3^. Alguns fatores explicam esse desequilíbrio da microbiota intestinal em pacientes com DRC, como a presença de uremia. O elevado nível de ureia plasmática faz com que ocorra influxo dessa molécula para o lúmen intestinal. As bactérias que expressam a enzima urease realizam a hidrólise da ureia em amônia, aumentando o pH intestinal, promovendo alterações no seu meio bioquímico e favorecendo o crescimento de determinadas bactérias em detrimento de outras^4^. Além disso, essa toxicidade causa a ruptura das *tight junctions* (junções de oclusão), permitindo a translocação dos padrões moleculares associados a patógenos (PAMPs), como os lipopolissacarídeos (LPS) – componentes da membrana das bactérias gram-negativas^5^. Adicionalmente, fatores como sedentarismo, estresse, ingestão de álcool, dietas, aditivos e agrotóxicos alimentares, uso de antibióticos, genética, edema, constipação, alterações no estado nutricional e algumas doenças alteram a microbiota intestinal desses pacientes^6,7^. O contexto sociocultural no qual o indivíduo está inserido também contribui para a caracterização da microbiota intestinal; dessa forma, a variação interindividual desse microbioma costuma seguir o padrão característico entre os indivíduos residentes da mesma localidade geográfica^8,9^.

Todos esses fatores podem alterar a microbiota dos pacientes com DRC, e essa alteração pode apresentar diversas consequências, incluindo o aumento do processo inflamatório. Isso ocorre porque tanto os componentes da microbiota quanto as toxinas produzidas por ela podem alcançar a corrente sanguínea e ativar fatores de inflamação que levam ao aumento da produção de citocinas pró-inflamatórias, como o fator de necrose tumoral-α (TNF-α) e a interleucina-6 (IL-6), por monócitos ativados e células endoteliais^10^.

Pesquisas afirmam que a dieta e o estilo de vida são os fatores mais influentes em relação à caracterização da microbiota intestinal e estão intimamente relacionados ao contexto sociocultural e à localização geográfica desses indivíduos^11,12^. Os estudos sobre microbiota na DRC têm focado tanto em avaliar o perfil microbiano de forma geral, bem como em avaliar intervenções para mitigar a disbiose, com o uso de prebióticos, probióticos ou simbióticos^13^. Porém, é necessário estudar também o perfil da microbiota nos diferentes cenários geográficos e culturais, que são marcadamente diferentes, como o Sul e o Sudeste do Brasil.

Considerando que a localização geográfica e o contexto sociocultural influenciam de maneira significativa a composição da microbiota, sobretudo por meio de diferenças ambientais, padrões dietéticos regionais, hábitos de vida e até variações nos cuidados relacionados à diálise, torna-se relevante investigar como esses fatores podem impactar pacientes com DRC. Elementos como disponibilidade e tipo de alimentos consumidos, práticas culinárias regionais e possíveis diferenças assistenciais entre unidades de HD podem modificar tanto o perfil microbiano quanto o estado inflamatório desses indivíduos. Diante da escassez de estudos que avaliem essas particularidades no cenário brasileiro, especialmente em regiões cultural e ambientalmente distintas como o Sul e o Sudeste, fez-se necessária a realização desta pesquisa, cujo objetivo central foi caracterizar e comparar o perfil microbiano intestinal e a inflamação de pacientes com DRC em HD dessas duas regiões.

## Métodos

### Desenho do Estudo

Estudo transversal que incluiu pacientes com DRC em HD (realizando três sessões de diálise por semana, com duração de 4 horas) residentes nas regiões Sul (Blumenau) e Sudeste (Rio de Janeiro) do Brasil. O estudo incluiu homens e mulheres com idade entre 18 e 75 anos que realizavam HD há pelo menos seis meses e eram atendidos pelo Sistema Único de Saúde (SUS). Foram excluídos gestantes, pacientes transplantados, pacientes com doenças hepáticas, autoimunes, infecciosas, câncer ou síndrome da imunodeficiência adquirida (AIDS), bem como pacientes em uso de drogas catabólicas, de suplementos prebióticos, probióticos ou simbióticos, ou que utilizaram antibióticos e anti-inflamatórios nos três meses anteriores ao início do estudo.

O presente estudo foi aprovado pelo Comitê de Ética em Pesquisa da Faculdade de Medicina da UFF-Niterói, Rio de Janeiro, Brasil (CAAE 47703315.6.0000.5243 e 03219618.3.0000.5243), e todos os pacientes deram seu consentimento informado antes de iniciar o estudo.

As variáveis demográficas e clínicas utilizadas foram obtidas diretamente dos prontuários médicos, garantindo a padronização das informações e a fidelidade aos registros originalmente documentados pela equipe assistencial. Esse procedimento permitiu reunir dados completos e consistentes sobre o perfil dos participantes, assegurando a qualidade e a confi­abilidade das informações empregadas nas análises.

### Análise da Ingestão Alimentar e Avaliação do Estado Nutricional

A ingestão alimentar foi avaliada utilizando o recordatório alimentar de três dias, abrangendo dois dias úteis (um com diálise e outro sem diálise) e um dia de fim de semana. As análises de ingestão de energia e macronutrientes foram calculadas por meio do software NutWin®. É importante ressaltar que os pacientes seguiam a prescrição dietética individualizada fornecida pelas clínicas de diálise.

O estado nutricional foi avaliado de acordo com o índice de massa corporal (IMC), obtido como a razão entre o peso e a altura ao quadrado, seguindo a classificação proposta pela Organização Mundial da Saúde^14^.

Para a avaliação da dose de diálise, o Kt/V foi calculado utilizando a fórmula de Daugirdas (segunda geração), que estima a eficiência da hemodiálise a partir das concentrações de ureia pré e pós-diálise, do tempo de tratamento e da variação de peso corporal durante a sessão^15^.

### Processamento das Amostras e Procedimentos Analíticos

A coleta de sangue foi realizada em um único momento, pela manhã, após jejum, antes de iniciar o procedimento de diálise e imediatamente após a punção da fístula arteriovenosa (FAV). O sangue foi coletado em tubos Vacutainer® contendo ácido etilenodiaminotetracético (EDTA −1,0 mg/mL) como anticoagulante. O plasma foi separado (15 min, 3500 rpm, 4 °C) e armazenado a −80 °C até a análise.

#### Parâmetros bioquímicos de rotina e citocinas inflamatórias

Os níveis séricos de parâmetros bioquímicos, como ureia, cálcio, albumina, glicose, fósforo, potássio, colesterol total, lipoproteína de alta densidade (HDL), triglicerídeos e proteína C-reativa (PCR), foram analisados usando kits comerciais da BioClin®, por meio de um analisador bioquímico automático, de acordo com as instruções do fabricante. Os valores de lipoproteína de baixa densidade (LDL) foram calculados utilizando a fórmula de *Friedewald* (1972): LDL = (TC – HDL) – (TG/5)^16^.

Para a mensuração da concentração plasmática da citocina inflamatória IL-6 e do TNF-α, foram utilizados kits comerciais de ensaio imunoenzimático (ELISA). As concentrações de IL-6 e TNF-α foram medidas no intervalo de 31–2000 pg/mL com kits específicos de ELISA sanduíche quantitativos (*PeproTech*, Inc.).

#### Peroxidação lipídica

A peroxidação lipídica foi utilizada como marcador de estresse oxidativo, sendo avaliada por meio das substâncias reativas ao ácido tiobarbitúrico (TBARS), pelo método de *Ohkawa* modificado. As amostras foram diluídas com ácido tiobarbitúrico (0,6% p/v), dodecil sulfato de sódio (SDS) (8,1% p/v) e ácido fosfórico (1% p/v) e, posteriormente, aquecidas a 95 °C por 60 min. Os microtubos foram centrifugados a 4000 rpm durante 20 min a 20 °C; o sobrenadante foi removido e a absorbância foi medida utilizando um leitor de microplacas Synergy H1M (Biotek) a 532 nm. Os níveis plasmáticos de TBARS foram expressos em nmol/mL.

#### Sequenciamento e análise da microbiota intestinal

Os pacientes receberam previamente coletores de fezes estéreis e um manual contendo instruções sobre a coleta, o armazenamento e o transporte do material. Eles foram orientados a manter as amostras de fezes no freezer durante a noite e a levá-las sob temperatura controlada. Após o recebimento por nossa equipe, as amostras foram armazenadas a -20 °C para análise futura. A extração de DNA foi realizada de acordo com as instruções do fabricante (*Quick-DNA Fecal/Soil Microbe DNA Miniprep Kit, Zymo Research*). As concentrações finais de DNA foram determinadas por quantifi­cação espectrofotométrica usando o *NanoDrop 2000* (*Thermo Fisher Scientific*, Waltham, MA, EUA).

A região V4 do gene 16S rRNA foi amplificada por PCR usando os primers 515F 5’-GTGYCAGCMGCCGCGGTAA-3’ e 806R 5’-GGACTACNVGGGTWTCTAAT-3’ (anexados com as *tags* universais Illumina) e submetida à termociclagem, composta por 3 min de desnaturação inicial a 94 °C, seguida por 32 ciclos de 45 s a 94 °C, 1 min a 50 °C e 90 s a 72 °C, com uma etapa de extensão final de 10 min a 72 °C. Os amplicons resultantes foram acoplados a *barcodes* e sequenciados na plataforma Illumina NovaSeq PE250 (0,1 M de leituras brutas por amostra), de acordo com as instruções do fabricante, pela empresa Novogene (Califórnia, EUA). No total, 30 amostras de dois grupos (HD e PD) foram sequenciadas por amplicon.

As leituras de sequenciamento do amplicon do gene 16S rRNA foram pré-processadas utilizando o pipeline USEARCH (v. 11). Isso inclui o agrupamento de amostras, mantendo uma *tag* de amostra exclusiva nos cabeçalhos dos arquivos brutos, seguido pela montagem/fusão de leituras de extremidade pareada, primer de corte e filtragem de qualidade. Além disso, as abundâncias foram calculadas para sequências exclusivas, e realizou-se o agrupamento em unidades de taxonomia operacional (OTUs, do inglês *operational taxonomic units*) com 97% de identidade, seguido por uma etapa de redução de ruído para filtrar quimeras, resultando em uma tabela de OTUs de raio zero (zOTUs). As tabelas de características e taxonomia, além dos metadados, foram exportadas como objetos *phyloseq* para análise posterior no R versão 4.1.2.

### Análise Estatística

Variáveis demográficas e clínicas de linha de base numérica contínua foram comparadas por meio do teste não paramétrico de *Mann–Whitney* U. Testes qui-quadrado foram utilizados para comparar as frequências relativas dos diferentes níveis de variáveis nominais/categóricas. Os níveis plasmáticos das variáveis estudadas foram logaritmizados (base 10) quando necessário, e as expressões de mRNA estudadas foram logaritmizadas (base 2). Correlações de *Pearson* ajustadas foram realizadas com resíduos de modelos de efeitos fixos lineares com a inclusão de variáveis de confusão, a saber, idade, sexo, IMC, tempo em diálise e Kt/V. Modelos de efeitos fixos lineares múltiplos avaliaram diferenças entre os grupos S e SE, incluindo as mesmas variáveis de confusão. Os gráficos apresentam os efeitos marginais médios estimados e seus intervalos de confiança de 95%.

Na análise da microbiota intestinal, para permitir a comparação em bases iguais entre os grupos S e SE, os dados foram rarefeitos para as análises de comparação a jusante. Dados rarefeitos geraram índices de diversidade α (número observado de zOTU e diversidade de *Shannon*). Dados brutos de zOTU foram normalizados com a biblioteca DESeq2 em R^17^ e utilizados para calcular a distância UniFrac ponderada, que foi visualizada por meio da análise de coordenadas principais (PCoA) com o pacote phyloseq implementado em R. A análise de variância multivariada permutacional (PERMANOVA) foi realizada na matriz de dados para comparar a estrutura ou a composição das comunidades microbianas. A análise multivariada permutacional de dispersão (PERMDISP) também foi realizada usando a função *‘*betadisper’ (implementada na versão *vegan* 2.6-4 da biblioteca) com 999 permutações.

As análises descritivas e de comparação de médias, bem como as análises de redundância e correlação de *Pearson*, foram realizadas em R, versão 4.1.2, usando as bibliotecas ‘emmeans’ versão 1.8.2 e ‘microViz’ versão 0.11.0. As relações entre zOTUs e grupos de amostras foram medidas por meio da biblioteca ‘indicspecies’ versão 1.7.14. Todos os gráficos e plotagens foram gerados utilizando as bibliotecas ggplot2 versão 3.4.0 ou TBtools^18^.

## Resultados

Um total de 30 pacientes foi incluído, sendo 14 no grupo S e 16 no grupo SE. A Tabela 1 mostra os dados demográficos, de ingestão alimentar e os parâmetros bioquímicos e inflamatórios dos pacientes incluídos no estudo. Os parâmetros demográficos e clínicos (idade, gênero, tempo de diálise, Kt/V e IMC) não apresentaram diferença estatística; no entanto, a ingestão de energia (p-valor = 0.029) e de carboidratos (p-valor = 0.017) foi maior no grupo Sudeste. Os pacientes da região Sul apresentaram maiores valores séricos de albumina (p-valor = 0.011) e potássio (p-valor = 0.011). Além disso, os pacientes da região Sul exibiram maiores valores plasmáticos de citocinas inflamatórias (Figura 1).

**Tabela 1:** Parâmetros demográficos, dietéticos, bioquímicos e inflamatórios de pacientes com DRC

Parâmetros	Grupo sul (n = 14)	Grupo sudeste (n = 16)	Valor de p
**Demográficos e Clínicos**			
Idade (anos)	58 (13,5)	57 (19)	0,691
Sexo [n (%)] (masculino/feminino)	7(50%)/7(50%)	8 (47,1%)/9 (52,1%)	1,000
Tempo de diálise (meses)	40,5 (27)	68 (79)	0,142
Kt/V	1,40 (0,2)	1,42 (0,18)	0,883
IMC (kg/m^2^)	24,8 (4,57)	24,4 (4,43)	0,592
**Dietéticos**			
Energia (kcal/dia)	1035,5 (368,7)	1499,3 (568)	**0,029**
Carboidrato (g/dia)	135,8 (46,0)	213,34 (89,8)	**0,017**
Proteína (g/dia)	53,7 (25,3)	74,2 (43,6)	0,161
Lipídio (g/dia)	35,4 (18,0)	40,3 (12,3)	0,487
Fibra (g/dia)	10,2 (6,4)	13,7 (5,6)	0,207
**Bioquímicos**			
Glicose (mg/dL)	97 (63,5)	100 (26)	0,682
Colesterol total (mg/dL)	163,5 (34)	153 (30)	0,706
Triglicerídeos (mg/dL)	159,5 (58)	114 (41)	0,108
HDL (mg/dL)	43,5 (6)	40 (8)	0,320
LDL (mg/dL)	78,4 (28)	57,0 (51)	0,511
Fósforo (mg/dL)	5,1 (0,9)	4,6 (1,3)	0,103
Potássio (mg/dL)	4,0 (0,2)	3,7 (0,4)	**0,011**
Cálcio (mg/dL)	8,0 (1,1)	8,9 (0,6)	0,062
Ureia (mg/dL)	130,5 (19,7)	140 (3,5)	0,180
Albumina (mg/dL)	4,0 (0,2)	3,7 (0,4)	**0,011**
**Inflamatórios**			
PCR (mg/dL)	4,7 (8,8)	6,5 (9,2)	0,580
TBARS (nmol/mL)	2,5 (2,4)	1,4 (4,1)	0,467

Abreviaturas – DRC: doença renal crônica; KT/V: eficácia da diálise; IMC: índice de massa corporal; HDL: lipoproteína de alta densidade; PCR: proteína C-reativa; TBARS: substâncias reativas ao ácido tiobarbitúrico. Notas – Os dados são apresentados como frequências absolutas (relativas) ou mediana (intervalo interquartil – IIQ); os valores de p foram estimados por testes qui-quadrado ou pelo teste não paramétrico de *Mann-Whitney* U.

**Figura 1:** Comparação dos níveis plasmáticos de marcadores inflamatórios. Foram observadas diferenças nos níveis plasmáticos de TNF-α (A) e IL-6 (B) entre os grupos Sul e Sudeste.

Notas – As distribuições de amostra de dados são apresentadas em *box-strip plots*. Em preto, o círculo central representa o efeito marginal médio para cada grupo estimado a partir de modelos lineares múltiplos de efeitos fixos, incluindo as variáveis de confusão idade, sexo, IMC, tempo em diálise e Kt/V. As barras horizontais pretas representam os intervalos de confiança de 95% do efeito marginal médio estimado por grupo.

### Estrutura e Diversidade da Microbiota Intestinal de Pacientes com DRC em Grupos Distintos de Diálise

A diversidade microbiana (índice de *Shannon*, Figura 2A), a riqueza (número observado de zOTUs, Figura 2B) e a estrutura da comunidade microbiana (distância UniFrac ponderada, Figura 2C), no nível zOTU, da microbiota intestinal de pacientes com DRC em HD residentes nas regiões Sul e Sudeste do Brasil não mostraram diferenças significativas.

**Figura 2:** Diversidade α e β e estrutura da comunidade microbiana da microbiota intestinal de pacientes com DRC em HD residentes nas regiões Sul e Sudeste do Brasil.

Notas – zOTUs observados (A), índice de *Shannon* (B), e comparação da estrutura da comunidade (C) (distâncias UniFrac ponderadas) usando análise de coordenadas principais (PCoA), mostrando a variabilidade percentual no microbioma causada pelos grupos.

A Figura 3 mostra os táxons microbianos mais abundantes nas 30 amostras analisadas. Os filos Firmicutes e Bacteroidetes e as famílias Lachnospiraceae e Ruminococcaceae foram os táxons mais abundantes encontrados em todas as amostras.

**Figura 3:** Composição microbiana intestinal de pacientes com DRC em HD residentes nas regiões Sul e Sudeste.

Notas – Gráfico de barras representando os filos (A) e as famílias (B) mais abundantes em cada amostra.

O correlograma (Figura 4) representa as correlações significativas (p < 0,05) entre os filos bacterianos e os marcadores inflamatórios (IL-6 e TNF-α). Embora a análise de diversidade α e β, bem como a estrutura da comunidade microbiana entre as duas regiões, não tenham apontado difer­enças, foram encontrados padrões de correlações distintos. No grupo Sudeste, observou-se que não houve correlação dos níveis plasmáticos de TNF-α com nenhum táxon analisado; entretanto, foi observada correlação negativa de IL-6 com Lentisphaerae e correlação positiva de IL-6 com Tenericutes. Já no grupo do Sul, não foi encontrada correlação dos níveis plasmáticos de IL-6 com nenhum táxon analisado, enquanto TNF-α correlacionou-se negativamente com Spirochaetae e positivamente com Fusobacteria.

**Figura 4:** Correlograma entre os filos bacterianos, táxons microbianos e marcadores inflamatórios.

Notas – Apenas as correlações significativas (p < 0,05) foram mostradas no gráfico: positivas (azul) e negativas (vermelho) de acordo com o teste de correlação linear de *Pearson* ajustado por idade, sexo, IMC e tempo em diálise, além de Kt/V.

## Discussão

Nosso estudo teve como objetivo caracterizar e comparar o padrão microbiano intestinal e o perfil inflamatório de pacientes em HD residentes nas regiões Sul e Sudeste do Brasil, e observou-se que esses pacientes apresentaram o mesmo padrão microbiano intestinal. No entanto, verificou-se que os pacientes da região Sul apresentaram um perfil inflamatório exacerbado quando comparados aos da região Sudeste.

O grupo Sudeste apresentou maior ingestão de energia quando comparado ao grupo do Sul, e esse dado foi reflexo do maior consumo de carboidratos. Porém, não foi observada correlação com a composição da microbiota intestinal. Vale ressaltar que a ingestão alimentar foi avaliada utilizando o recordatório alimentar de três dias. Embora esse método forneça informações sobre o padrão alimentar da população em estudo, essa ferramenta apresenta algumas limitações; dessa forma, ela não fornece estimativa segura da absorção de nutrientes^19^. Contudo, é muito provável que esses indivíduos apresentem baixa variabilidade de consumo alimentar, visto que nossa amostra foi composta por pacientes atendidos pelo SUS, que majoritariamente pertencem a estratos socioeconômicos mais baixos, com menor poder aquisitivo e acesso limitado a alimentos variados e de qualidade nutricional.

Pacientes do Sul apresentaram aumento dos níveis séricos de albumina, comparados ao grupo Sudeste. Uma possível explicação para esse resultado é a diferença no acompanhamento nutricional desses pacientes. Nerbass et al.^20^ constataram que, dentre as regiões do Brasil, a que fornece suplementação alimentar gratuita com maior frequência para pacientes em diálise foi a região Sul (71%), seguida da região Nordeste (55%), Norte (50%), Centro-Oeste (49%) e Sudeste (40%).

Neste estudo, utilizamos sequenciamento de alto rendimento para comparar a microbiota intestinal de pacientes com DRC em HD residentes nas regiões Sul e Sudeste. O objetivo foi elucidar se a cultura local, juntamente com os fatores dietéticos, influencia a composição microbiana intestinal, identificando possíveis padrões microbianos intestinais e facilitando a abordagem dietoterápica desses pacientes. Refutando nossa hipótese inicial, a composição da microbiota intestinal em pacientes com DRC submetidos à HD, residentes nas diferentes regiões do Brasil avaliadas (Sul x Sudeste), não diferiu. Um padrão bacteriano marcante foi observado em ambos os grupos, com abundância predominante dos filos Firmicutes e Bacteroidetes e das famílias Lachnospiraceae e Ruminococcaceae.

Até o momento, nenhum estudo diferenciou a microbiota de pacientes com DRC em diferentes regiões do Brasil, assim como não avaliou os parâmetros inflamatórios. É importante ressaltar que nossa análise observou os táxons em nível de filo e família; portanto, não podemos afirmar a superioridade de um padrão microbiano sobre o outro em níveis taxonômicos de gênero ou espécie, visto que a mesma família pode conter linhagens bacterianas comensais ou patogênicas. Dessa maneira, estudos utilizando abordagens metagenômicas e sequenciamento de leituras longas de amplicon devem ajudar a elucidar, no futuro, os padrões de distribuição do microbioma em diferentes pacientes. Além disso, a disponibilidade de substrato pode interferir na via de metabolização das bactérias intestinais, de forma que táxons bacterianos proteolíticos fecais e, portanto, geradores de toxinas urêmicas, também podem gerar compostos fenólicos, dependendo do substrato ofertado^21,22^.

Alguns estudos relatam alterações da microbiota intestinal em pacientes com DRC. O primeiro que revelou um perfil alterado da microbiota intestinal nesse grupo de pacientes, com aumento do crescimento de bactérias dos gêneros *Streptococci, Lactobacilli* e *Bacteroides*, foi realizado em 1978. A hipótese proposta pelos autores foi que a uremia ocasionava redução do trânsito intestinal e comprometimento do sistema imune, propiciando o crescimento de algumas bactérias^23^. Vaziri et al.^24^ observaram que pacientes com DRC apresentavam diferença significativa em 190 OTUs quando comparados a indivíduos saudáveis. Esses indivíduos exibiram expansão significativa de bactérias intestinais com enzimas formadoras de toxinas urêmicas, associada à redução de famílias bacterianas que levam à formação de ácidos graxos de cadeia curta (AGCC). Esse perfil caracteriza uma composição desfavorável, tendo em vista os efeitos deletérios das toxinas urêmicas e os efeitos benéficos dos AGCC^7,24,25^. Em pacientes com DRC, o cólon torna-se a principal via secretora de ácido úrico, bem como de oxalato; esse mecanismo explica a expansão de espécies bacterianas produtoras de uricase^26^. A microbiota de pacientes com DRC em HD parece ser diferente daquela de controles saudáveis, apresentando maior abundância dos filos bacterianos Actinobacteria, Firmicutes e Proteobacteria, em comparação com controles saudáveis^24^. A população microbiana intestinal também é diferente em pacientes submetidos à DP, em comparação com controles saudáveis, com menor abundância de *Bifidobacterium catenulatum*, *Bifidobacterium longum*, *Bifidobacterium bifidum*, *Lactobacillus plantarum*, *Lactobacillus paracasei* e *Klebsiella pneumoniae* ^27^. Dessa forma, pacientes com DRC parecem ter maior abundância de bactérias que expressam urease, uricase e enzimas formadoras de indol e p-cresol^28^.

Nosso estudo encontrou padrões de correlações distintos. No grupo Sudeste, foi encontrada correlação negativa de IL-6 com Lentisphaerae e correlação positiva de IL-6 com Tenericutes. Outro trabalho realizado pelo nosso grupo encontrou correlação positiva de Fusobacteria com a toxina urêmica indoxil sulfato (IS), e negativa com Lentisphaerae em pacientes em HD^29^. Zhang et al.^30^ revelaram que o filo Lentisphaerae apresentou correlação negativa com o risco de sepse. Em contrapartida, o filo Tenericutes foi positivamente relacionado ao risco de sepse em indivíduos com menos de 75 anos.

Além disso, Nagu et al.^31^ detectaram que táxons intestinais, como Actinobacteria, Bacteroidetes, *Escherichia coli*, Firmicutes, Proteobacteria, Tenericutes e Verrucomicrobia, são conhecidos por desempenharem papel crucial na patogênese da doença de Alzheimer, uma vez que contribuem para a neuroinflamação e outros processos inflamatórios, prejudicando os processos de neurodesenvolvimento.

Já no grupo Sul, os valores de TNF-α correlacionaram-se positivamente com Fusobacteria e negativamente com Spirochaetae. Alguns estudos demonstram a influência de Fusobacteria *s*obre a etiologia do câncer colorretal. O mecanismo para tal associação é complexo, mas pesquisas demonstram que *Fusobacterium nucleatum* promove inflamação intestinal, estimulando a secreção das citocinas pró-inflamatórias interleucina-8 (IL-8) e fator de necrose tumoral (TNF) por meio de vesículas de membrana externa que ativam *Toll-like receptor 4* (TLR-4) e NF-κB^32,33^. De forma geral, Fusobacteria é um anaeróbio Gram-negativo que desempenha papel fundamental em biofilmes orais e está envolvido em doenças periodontais e extraorais, sendo o câncer colorretal a mais proeminente, caracterizando-se como um táxon promotor de inflamação^34,35,36^.

Mais estudos são necessários para compreender e elucidar a correlação negativa que encontramos de Spirochaetae com TNF-α, visto que esse táxon possui três gêneros que causam infecção humana: *Treponema* spp., que causa sífilis; *Borrelia* spp., responsável pela doença de Lyme, e *Leptospira*, o agente causador da leptospirose^37^. A fisiopatologia dessas infecções é complexa, e entender tais mecanismos pode esclarecer nossos resultados.

Um estudo revelou que a artrite de Lyme ocasiona uma resposta imune pró-inflamatória excessiva, caracterizada pela produção de IFN-γ e por quantidades inadequadas da citocina anti-inflamatória IL-10, promovendo dano tecidual, vascular, processos autoimunes e citotóxicos, bem como proliferação de fibroblastos e fibrose^38^. Dessa forma, mesmo que Spirochaetae seja um táxon que abrange espécies causadoras de doenças inflamatórias, talvez a fisiopatologia não promova aumento de TNF-α, mas sim de outras citocinas inflamatórias, como o IFN-γ.

De forma geral, apesar de nosso estudo não ter identificado diferenças significativas na estrutura global da microbiota entre as duas regiões analisadas, conforme demonstrado pelas métricas de diversidade α e β, observamos alguns padrões distintos de correlação entre filos bacterianos e marcadores inflamatórios, como discutido anteriormente. Entretanto, essas associações devem ser interpretadas com cautela. Como não houve diferenças claras na composição microbiana entre os grupos, é possível que os padrões observados reflitam variabilidade intragrupo ou confusão residual não capturada pelas análises. Assim, embora os achados levantem hipóteses interessantes sobre possíveis interações entre a microbiota e as vias inflamatórias, eles não permitem inferir relações causais ou diferenças biológicas robustas entre as regiões. Estudos com maior poder amostral, análises funcionais e controle mais amplo de potenciais confundidores são necessários para confirmar ou refutar essas observações.

Há escassez de estudos comparativos que avaliem simultaneamente a microbiota intestinal e os marcadores inflamatórios em pacientes com DRC em HD residentes em diferentes regiões do Brasil. Apesar de existirem estudos nacionais focados em caracterizar a disbiose associada à DRC e suas correlações com toxinas urêmicas, inflamação e dieta, esses trabalhos têm sido conduzidos predominantemente em coortes localizadas, sem análises multicêntricas que permitam identificar variações geográficas, o que torna este estudo relevante e original.

Este trabalho apresenta algumas limitações, como a ausência de aplicação de um formulário para identificar variáveis socioeconômicas, embora soubéssemos que 100% da nossa amostra era composta por pacientes atendidos pelo SUS. Outro fator importante é a avaliação do uso de medicamentos específicos; no entanto, tivemos cuidado na seleção dos pacientes, excluindo indivíduos em uso de drogas catabólicas, suplementos prebióticos, probióticos ou simbióticos, bem como aqueles que utilizaram antibióticos e anti-inflamatórios nos três meses anteriores à coleta de sangue.

Embora nossas análises não tenham evidenciado diferenças marcantes na diversidade ou na estrutura da microbiota entre as regiões, os padrões regionais distintos de associação entre táxons bacterianos e marcadores inflamatórios sugerem que o contexto ambiental e dietético pode influenciar, de maneira diferenciada, a interação microbiota–inflamação em pacientes em hemodiálise. Esse tipo de informação tem potencial para orientar o desenvolvimento de abordagens de nutrição de precisão, que considerem não apenas a composição microbiana, mas também suas inter-relações funcionais com o estado inflamatório. Assim, compreender como os perfis regionais moldam essas interações pode contribuir para o desenvolvimento de estratégias mais individualizadas de modulação da inflamação, oferecendo caminhos promissores para intervenções focadas na melhora do prognóstico desses pacientes.

## Conclusão

Nosso estudo observou que, embora pacientes com DRC em HD residentes nas regiões Sul e Sudeste apresentem padrões similares de diversidade e estrutura das comunidades microbianas intestinais, o grupo do Sul apresentou maior concentração de marcadores inflamatórios (TNF-α e IL-6). É importante ressaltar que nosso estudo foi pioneiro nesse campo e, portanto, estudos adicionais são necessários para contribuir com nossos achados.

## Data Availability

Os autores confirmam que os dados que sustentam os resultados deste estudo estão disponíveis no próprio manuscrito.
